# Relative testis size is associated with vagina length but not sperm storage traits in Galliformes

**DOI:** 10.1093/evlett/qraf035

**Published:** 2025-10-03

**Authors:** Katherine Assersohn, Nicola Hemmings

**Affiliations:** School of Biosciences, Alfred Denny Building, University of Sheffield, Sheffield, S10 2TN, United Kingdom; School of Biosciences, Alfred Denny Building, University of Sheffield, Sheffield, S10 2TN, United Kingdom

**Keywords:** female fertility, sexual selection, sperm competition, sperm morphology, sperm storage tubule

## Abstract

Post-copulatory sexual selection, comprised of sperm competition and cryptic female choice, is a powerful evolutionary force that can drive the rapid diversification of reproductive traits across taxa. In birds, the female reproductive tract provides the arena for post-copulatory sexual selection, yet we lack a comprehensive understanding of the female specific processes that shape the evolution of sexually selected traits. Here, we use a comparative approach to explore the relationships between female reproductive tract morphology, sperm competition intensity, and sperm traits across Galliformes. Accounting for phylogenetic and allometric relationships, we find that species with relatively larger testes for their body size—a proxy for intense sperm competition—have relatively longer vaginas, suggesting that important co-evolutionary dynamics exist between male and female reproductive physiology. Surprisingly, we find no link between sperm length and sperm storage tubule morphology, challenging existing predictions. Our findings suggest that the vagina has a significant but currently overlooked influence on post-copulatory processes and emphasizes the need to better integrate female morphology into models of sexual selection.

## Introduction

Post-copulatory sexual selection can drive the rapid evolution of morphological, physiological, and behavioral reproductive traits (Birkhead & Pizzari, 2002). When females mate with multiple males, sexual selection can continue post-insemination through both sperm competition—where ejaculates from different males compete for fertilization of the ova (Parker, 1970)—and cryptic female choice—where females bias paternity toward sperm from preferred males (Eberhard, 1996).

The female reproductive tract provides the arena for post-copulatory processes, and sperm selection is expected to be strongest in the vagina (Bakst et al., 1994; Birkhead & Brillard, 2007; Steele & Wishart, 1992). In birds, the vagina is so effective at reducing sperm numbers that less than 1% of ejaculated sperm make it through to the site of storage (Bakst et al., 1994; Birkhead & Brillard, 2007). This rapid post-copulatory sperm loss is at least in part due to the hostile nature of the vagina, in which mechanical flushes and muscular contractions (Bakst et al., 1994; Matsuzaki et al., 2015; Pizzari & Birkhead, 2000), anti-sperm compounds (Huang et al., 2016), immunological activity (Bakst, 2011; Bakst et al., 1994; Higaki et al., 1995; Yoshimura et al., 1997), and even its anatomical structure (Brennan et al., 2010) can act to impede, eject, or incapacitate sperm. The vagina therefore provides a selective environment in which males/sperm better able to overcome these obstacles can achieve greater fertilization success, which should, in turn, result in the co-evolution of male and female adaptations for control over paternity (Birkhead & Pizzari, 2002; Brennan et al., 2007). This has been most well-studied from the male perspective: for example, when sperm competition is intense, selection favors increased investment in testis mass, because larger testes (relative to body mass) are associated with increased sperm production (Lüpold et al., 2009b; Ramm & Stockley, 2010). This relationship is so well established that testis mass is commonly used as a proxy for sexual selection intensity across taxa. In some taxa (e.g., mammals and insects), there is increasing evidence that post-copulatory selection can also result in sexually antagonistic co-evolution between male reproductive traits and female reproductive tract anatomy (Arnqvist & Rowe, 2002; Brennan & Prum, 2015; Orr & Brennan, 2015). For example, in response to sexual conflict over mating rate, male water striders have evolved clasping armaments, while females have evolved antagonistic anti-clasping traits (Arnqvist & Rowe, 2002). Garter snakes (*Thamnophis sirtalis*) present a more subtle example; while the male has evolved hemipene spines that can control mating rate, females can terminate copulation through muscular contraction, likely due to thickened vaginal walls (Brennan & Prum, 2015; Friesen et al., 2014). Few studies have explored sexually antagonistic coevolution in genital morphology in birds, with the notable and striking exception of the anticlockwise corkscrew penis and clockwise morphology of the vagina in waterfowl (Brennan et al., 2007).

Females that mate with multiple males are expected to develop sperm selection mechanisms, but they must balance this against the need for sufficient sperm at the time of ovulation (Assersohn et al., 2021; Hemmings et al., 2015). Unlike most mammals—where insemination must be precisely timed with the release of ova—female birds can store sperm from a single copulation within specialized structures known as sperm storage tubules (SSTs) (Das et al., 2008). SSTs, located at the distal end of the vagina in a region known as the utero-vaginal junction (UVJ), are not only essential for ensuring sperm are available for sequential ovulations but may provide an additional opportunity for female control over paternity through selective sperm storage (Firman et al., 2017; Hemmings & Birkhead, 2017; Ito et al., 2011; Mendonca et al., 2019; Sasanami et al., 2015; Steele & Wishart, 1996).

While we still do not fully understand the mechanisms by which SSTs accept, maintain, and release sperm, research suggests they are highly specialized and under fine temporal and possibly nervous control (Khillare et al., 2018). Recent evidence also suggests that SST morphology can be highly variable across some species (Assersohn et al., 2024). Sperm are the most diverse cell types in the animal kingdom (Pitnick et al., 2009), so it stands to reason that SSTs may have co-evolved with the sperm cells they store. However, despite some evidence that SST length correlates with sperm length across passerines (Birkhead & Møller, 1990), we know little about the relationship between SST morphological diversity, sperm traits, and post-copulatory sexual selection. Since SSTs play an essential role in the post-copulatory fate of sperm, understanding SST function is vital to our understanding of sexual selection. Additionally, whilst it has been acknowledged that SSTs may facilitate cryptic female choice, the relative contribution of SSTs to the total selective potential of the vagina is unknown.

Here, we conduct a comparative analysis (controlling for phylogeny and body mass) across the Galliformes, a diverse group of heavy-bodied land fowl, to explore the relationships between relative testis size, vaginal and SST traits, and sperm morphology. Given that the vagina provides a selective environment for sperm, and more intense selection should drive (or be driven by) increased sperm production, we first test the prediction that relative testis size correlates positively with relative vagina length. Second, we assess whether SSTs play a functional role in post-copulatory sperm selection by testing whether interspecific variation in SST traits is associated with (i) post-copulatory sexual selection intensity and (ii) sperm morphology.

## Results and discussion

**Figure 1.**
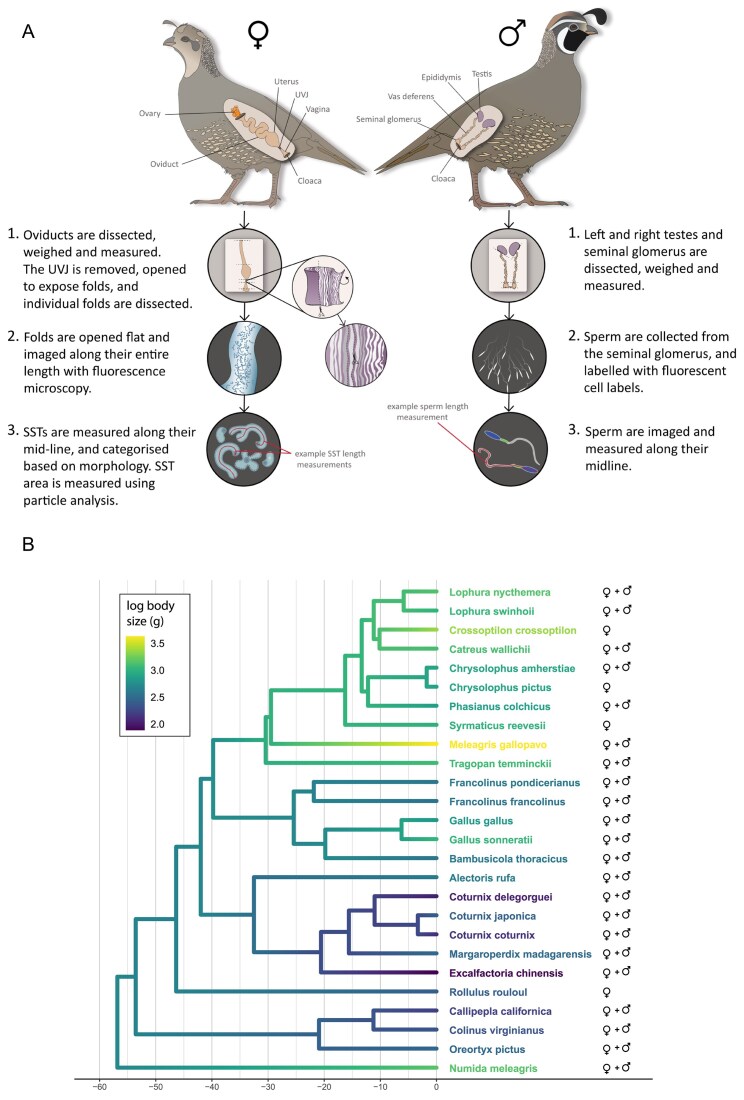
(A) Graphical summary of oviduct, testes, sperm storage tubule (SST), and sperm analysis procedures, showing steps from dissection to data collection for each sex. Full details of materials, reagents, and equipment are in the main text and supplementary material. Some elements of this figure are adapted from Assersohn et al. (2024)—see Figure 1 therein for a more detailed depiction of SST dissection. Graphic produced in Illustrator v29.2.1. (B) Galliformes phylogeny for the 26 species analysed in our dataset, with estimated ancestral state for body size (log) incorporated. Ancestral state estimates were generated using the R package *phytools* (Revell, 2024) with the Brownian motion model. Species for which we had oviduct samples are indicated by ♀, and those for which we also had testis and sperm data are indicated by ♂.

We dissected and measured the oviduct of 26 species of Galliformes, extracting and imaging the UVJ and SSTs (Figure 1). We also collected and measured testes and sperm from 20 species and extracted data for another 2 species from Liao et al. (2019), resulting in testes and sperm length measurements for 22 species (Figure 1; see also the *Methods* section).

### Vagina length as an indicator of post-copulatory sexual selection intensity

**Figure 2.**
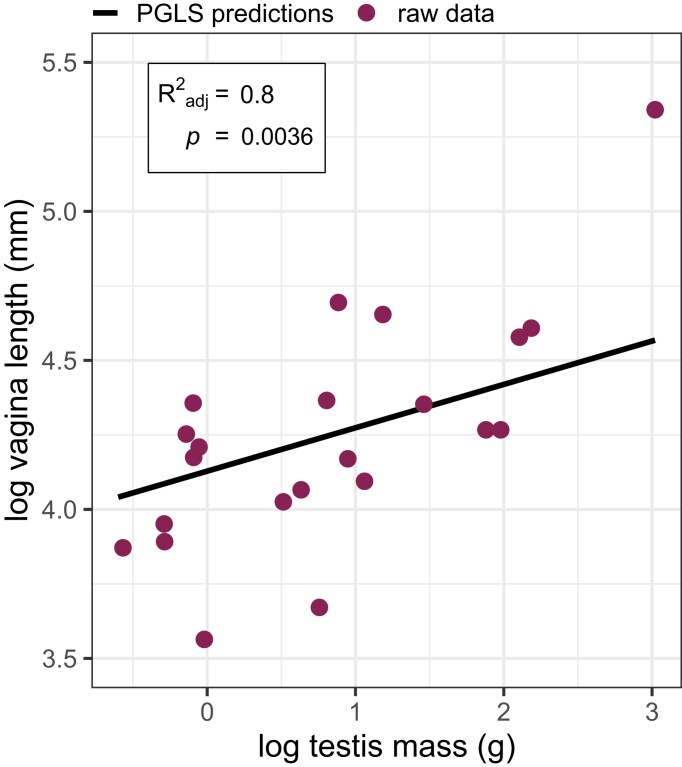
The relationship between vagina length and testis mass across 22 species of Galliformes. Dots represent raw data points (each a distinct species), and the solid line gives the predictions from the phylogenetic generalized least squares (PGLS) model, which corrects for phylogeny and body mass. For the sake of plotting, predictions were calculated on data with body mass held constant at the mean to remove variation as a result of allometric relationships. Adjusted *R*^2^ and *p*-values from the PGLS model are provided.

After accounting for variation associated with body mass and phylogenetic relationships, we found vagina length and testis mass were highly positively correlated, such that in species where males had relatively large testes, females also had relatively long vaginas (λ = 0; *R*^2^_adj_ = 0.8, *F* = 44, *df* = 2, 19, *p* = .0036, *n* = 22) (Figure 2). We therefore included vagina length as a proxy for post-copulatory sexual selection intensity in subsequent models (where relevant).

Relative testis mass is typically strongly associated with the amount of sperm producing tissue in the testes (Lüpold et al., 2009a) and increases with sperm competition intensity. Our results suggest that a similar association exists between vagina length and sperm competition intensity. The vagina is typically hostile to sperm, removing, incapacitating, or impeding their progress. Increased vagina length is likely to extend the exposure of sperm to such selective processes, intensifying selection for traits that counteract sperm loss, such as higher sperm concentration, speed, or resilience. This, in turn, should select for enhanced effectiveness of excess sperm removal by the female to ensure only the highest quality sperm are stored. The expected outcome of these processes is that relative vagina length should be positively associated with sperm competition intensity. To our knowledge, this is the first time this relationship has been demonstrated empirically. A few studies in mammals find evidence that sperm production is positively associated with oviduct length (Anderson & Dixson, 2006; Gomendio & Roldan, 1993; Weber & Fisher, 2023), but since the reproductive tract beyond the vagina is likely benign (i.e., non-selective), whole oviduct length is far less biologically relevant than vagina length when considering post-copulatory selective processes.

Our findings have several important potential implications: (i) relative vagina length and relative testis mass likely co-evolve; (ii) vaginal sperm selection is a powerful selective force and may represent a cross-taxa female adaptation for post-copulatory control; (iii) relative vagina length can be used as a proxy for sperm competition intensity (as it has in this study).

### The relationship between sexual selection intensity and sperm storage traits

**Figure 3.**
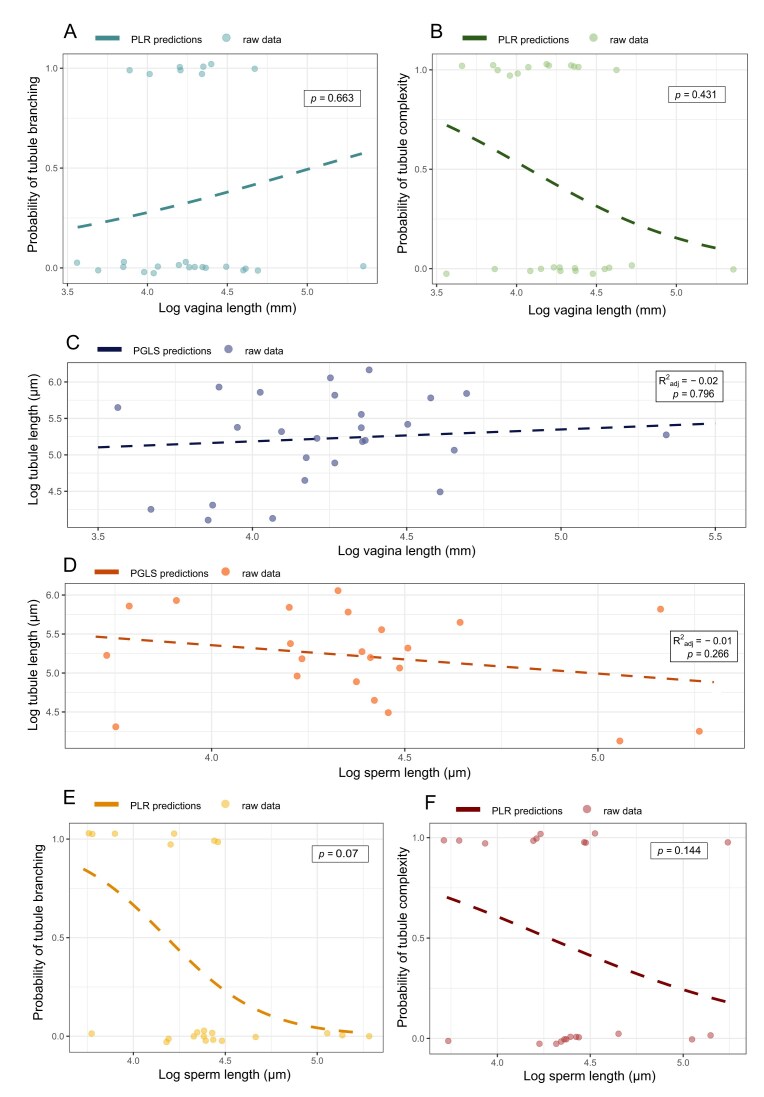
(A) and (B) Variation in the probability that a species will have branched (A) or complex (B) tubules [across the entire utero-vaginal junction (UVJ)] in relation to vagina length. *p-*values from the phylogenetic logistic regression (PLR) models are presented, and all points are jittered slightly to avoid overlaying points and aid visualization (*n* = 26). (C) and (D) The relationship between average tubule length (within the region of highest tubule density) and vagina length (*n* = 26) (C), and sperm length (*n* = 22) (D). *p-*and adjusted *R*^2^ values from the phylogenetic generalized least squares (PGLS) models are presented. (E) and (F) Variation in the probability that a species (*n* = 22) will have branched (E) or complex (F) tubules (across the entire UVJ) in relation to sperm length. *p-*values from the PLR models are presented. All points are jittered slightly to avoid overlaying points and aid visualization. For all plots, dots represent the raw data points, and the dashed line gives the (non-significant) predictions from either PLR or PGLS regression models, correcting for phylogeny and body mass. For the sake of plotting, all predictions were calculated on data with body mass held constant at the mean.

In addition to the relationship between vagina length and relative testis mass, we also investigated the potential drivers of female sperm storage trait variation. We found that SST tissue area (i.e., sperm storage capacity) was positively correlated with SST length, albeit with a moderate amount of unexplained variation (λ = 0, *R*^2^_adj_ = 0.51, *F* = 13.9, *df* = 2, 23, *p* = .0274, *n* = 26) (supplementary Figure S1). To avoid multicollinearity issues, we included only SST length in our models, since it was the more repeatable measure (see supplementary material). After accounting for variation associated with body mass and phylogeny, relative vagina length was not significantly correlated with either the degree of SST branching (α = 0.88, *Z* = 0.44, *p* = .663, *n* = 26) (Figure 3A), tubule complexity (α = 0.95, *Z* = −0.79, *p* = .431, *n* = 26) (Figure 3B), or tubule length (λ = 0, *R*^2^_adj_ = −0.02, *F* = 0.72, *df* = 2, 23, *p* = .796, *n* = 26) (Figure 3C). Since SST length and storage capacity (SST tissue area) were significantly positively correlated across species (which is not surprising given that longer tubules necessarily contain more tissue per unit^2^), we assume a lack of an association with SST area as well.

The lack of a clear relationship between post-copulatory sexual selection intensity and SST morphology is surprising, but it remains possible that SSTs are involved in post-copulatory processes unrelated to their size and capacity. SSTs could influence sperm selection through (i) dynamic changes to tubule morphology; (ii) variation in the internal molecular processes that control sperm quiescence and maintenance; or (iii) variation in the timing of sperm acceptance and release. There is evidence that SSTs contain gate-like entrances, capable of plastic contraction (Freedman et al., 2001; Hemmings & Birkhead, 2017; Mendonca et al., 2019), providing a potential mechanism for probable selection previously observed in/by SSTs (Hemmings & Birkhead, 2017; Ito et al., 2011; Sasanami et al., 2015; Steele & Wishart, 1996).

If SSTs can contract or relax along their entire length, SST morphology could also theoretically change through time. The presence of apparent coiled and peculiar “expanded and bulbous” tubules in some species of Galliformes (Assersohn et al., 2024) supports the theory that SSTs are capable of contraction and relaxation along their length (Mendonca et al., 2019), and there is also evidence that individual tubules are innervated (Freedman et al., 2001). Developing novel methods for observing SST function, particularly in response to ejaculations from different males, will be important for future work exploring the role of SSTs in post-copulatory sexual selection.

Variation in the structure of vaginal tissue underlying the SSTs may also play a role in sperm selection. For example, UVJ tissue in Galliformes commonly contains groove-like “channels” that appear to terminate at the SST region and, in some cases, transition directly into tubules (Assersohn et al., 2024). These channels may facilitate the differential transport or storage/acceptance of sperm [e.g., from different males (Hemmings & Birkhead, 2017)]. We focused on measuring SST morphology and storage capacity in the region of highest tubule density, reasoning that this should capture the most functionally relevant tubules and ensure consistent comparisons across species (see the *Methods* section). However, SST function may also vary spatially within the UVJ, and future work could test whether such variation plays a role in post-copulatory processes.

It is possible that the lack of a relationship between SST morphology and sperm competition intensity simply indicates that SSTs are not a key site of sperm selection, contrary to predictions (Birkhead, 2000; Briskie & Montgomerie, 1993; Eberhard, 1996; Hellriegel & Ward, 1998). By the time sperm reach the SSTs, they may have already undergone rigorous selection and therefore represent the “fertilizing set”—a subset of the inseminated sperm population of the required “quality” for fertilization (Cohen & Tyler, 1980). Consistent with this hypothesis, experimental evidence in poultry shows that (a) the number of sperm stored in SSTs is strongly correlated with the number that reach the ovum (Brillard & Bakst, 1990), and (b) dead sperm inseminated beyond the vagina reach the site of fertilization in as great numbers as living sperm but never reach the site of fertilization if inseminated into the vagina (Allen & Grigg, 1957). It is widely accepted that morphologically abnormal sperm are unlikely to enter SSTs (although worth noting that this assumption is based on fairly limited evidence); (Bakst et al., 1994; Ogasawara et al., 1966; Sasanami, 2017), suggesting they may be selectively removed before reaching/entering the SSTs.

### The relationship between sperm storage morphology and sperm length

We found no evidence for a relationship between sperm length and SST morphology, including SST length (λ = 0, *R*^2^_adj_ = −0.01, *F* = 0.93, *df* = 2, 19, *p* = .266, *n* = 22) (Figure 3D); the degree of SST branching (α = 0.02, *Z* = –1.80, *p* = .07, *n* = 22) (Figure 3E); or tubule complexity (α = 0.93, *Z* = −1.46, *p* = .144, *n* = 22) (Figure 3F). Previous work in passerines found sperm length was positively correlated with SST length and negatively correlated with SST numbers across 20 species (Briskie & Montgomerie, 1993). Our results are not entirely comparable, since the earlier study classified each individual branch of an SST as a separate tubule, whereas we consider branched tubules as one long tubule of greater total length. We believe our approach is more functionally appropriate as a measure of tubule capacity, since while a branched tubule has multiple blind ends, it has only one entrance, meaning the function (acceptance and release of sperm) between branches is non-independent. Briskie & Montgomerie (1993) suggested there may be a co-evolutionary relationship between sperm length and SST length, such that protection of sperm by the SST is maximized when sperm “fit” well in the tubule. However, we do not believe this is likely in Galliformes, given that SSTs are generally many times larger than sperm. Galliformes also vary significantly in physiology and body size, possibly allowing greater scope for variation in reproductive structures compared to passerines. Galliformes, unlike passerines, also possess a “non-intromittent phallic rudiment,” the function of which is unknown (Herrera et al., 2013). However, while it is possible that this may aid in directing ejaculate into the vagina, there is no evidence that it deposits sperm further in and thus is unlikely to influence sperm storage (Brennan, 2013; Herrera et al., 2013). Our contrasting findings with the Briskie & Montgomerie (1993) study may also indicate that the co-evolutionary dynamics of post-copulatory sexual selection differ across bird groups. Future work exploring these relationships across other groups will be important for testing these hypotheses.

An alternative driver of variation in SST morphology and tissue area could be the duration that sperm reside in storage. Sperm storage duration varies considerably between species: from 5–10 days in Japanese quail (*Coturnix japonia*) to 14–21 days in chickens (*Gallus gallus domesticus)* and up to 15 weeks in turkeys (Birkhead & Møller, 1990; Sasanami, 2017). Females that store sperm for a longer period might require: (i) tubules with more protective qualities (such as increased length or shape complexity) to prevent sperm loss, or (ii) greater storage capacity due to passive sperm loss. However, passive sperm loss through extended storage may be counteracted by increased sperm production (Immler et al., 2007; Liao et al., 2019); in pheasants, for example, sperm storage duration is positively associated with increased sperm production (Liao et al., 2019) and negatively with sperm length (Immler et al., 2007; Liao et al., 2019), but sperm length and number do not trade-off, and there is no association between sperm morphometry and sperm competition risk.

One limitation of our approach is that we lack information on intraspecific variation, since we were only able to source one female per species for sampling. For some species (but not all, and not enough for inclusion in analysis), we were able to sample multiple individuals, anecdotally observing that between-individual trait differences appeared to be relatively small. However, knowledge of intra-specific variation in SST density and morphology is currently limited across all species, and this warrants further research. We also acknowledge that our limited sample sizes, particularly for analyses including testis mass and sperm length data, may mean individual data points hold more influence over our results and increase uncertainty. Large datasets in comparative analyses are difficult to obtain, particularly those that involve complex dissection of internal tissues, but exploring these relationships across a wider range of species would no doubt be fruitful.

## Summary

The coevolutionary dynamics between male and female reproductive structures remain largely understudied across taxa. Most research on genital evolution focuses on the large diversity in male genitalia and sperm traits, overlooking the diversity in female traits (Ah-King et al., 2014). Our results add to growing evidence that female genital tracts are variable, complex, and may covary with male genital traits in both birds and other taxa (e.g., Brennan & Prum, 2015; Brennan et al., 2007; Dallai et al., 2021; Freedman et al., 2001; Greenwood et al., 2022; Rönn et al., 2007). Further comparative analyses, using consistent and comparable methodologies, as well as the development of novel approaches for examining SST function in vivo, will be important for teasing apart differences between species and groups and the role of SSTs in post-copulatory sexual selection. The mechanisms of sperm selection within the female reproductive tract are clearly complex and likely driven by a host of possibly interactive processes that are difficult to identify due to their cryptic nature. We nevertheless urge that future work considers female processes and physiology when exploring post-copulatory sexual selection mechanisms. We have suggested several explanations for our findings, including the possibility that SST morphological complexity is associated with other mechanisms of post-copulatory sexual selection, including sperm storage duration and functional variation in sperm acceptance and release from storage. These represent exciting avenues for further research.

## Methods

### Oviduct and testis dissections

All birds used in this study were initially collected as part of an earlier project, obtained already deceased in 2016 from a licensed pheasant breeder disposing of excess stock. Testes and oviduct samples were preserved on site within 30 min of each bird being killed (Figure 1). Since the oviduct is known to regress outside of the reproductive period, females were only included if they were in breeding condition (demonstrated by the presence of an ovum in the oviduct, or a hierarchy of developing ova in the ovary), and male and female birds were all confirmed first-year breeders. Prior to culling, males and females were housed in small, mixed sex flocks, allowing for natural and unrestricted copulations, and females were dissected within 2 days of commencing egg-laying. We confirmed the fertility of previous eggs to ensure females had been recently successful in accepting and storing sperm. We also confirmed that males had well developed testes with sperm present in their seminal glomera at the time of dissection.

We measured body mass and oviduct traits of a single female per species. Whole oviducts were removed intact, unravelled, and stripped of connective tissue, then cleaned in phosphate-buffered saline (PBS). The wet mass was taken after briefly dabbing off excess liquid. Oviducts were then pinned in a shallow wax-based tray, photographed, and measured. Measurements were taken for the entire length, as well as each individual section, before being transferred into a deeper tray to be pinned and submerged in fixative (10% formalin solution) for at least 48 hr. We chose to measure length because the folded and somewhat channelled nature of the vaginal epithelium, and the relative absence of a wide open lumen (personal observations), make alternative metrics such as volume or surface area impractical. Moreover, length likely provides the more biologically relevant measure, given evidence in mammals indicating that sperm tend to travel in close association with the epithelial surface rather than swimming freely (Suarez, 2016).

A segment of the vagina containing the UVJ was then cut away, and individual UVJ folds were dissected and examined using fluorescence microscopy to measure SST length and area, as described in Assersohn et al. (2024). Each UVJ fold sample was also categorized by the presence or absence of different tubule morphotypes. Tubule morphotypes included “straight unbranched,” “straight branched,” or “complex” types, according to the categorization criteria in Assersohn et al. (2024) (see also supplementary material for full details of the UVJ dissection, imaging, and categorization). We also measured the body mass, testis mass, and sperm morphology of between 1 and 3 males (average 2.4) per species (see dataset for full details of the number of individuals sampled per species (Assersohn & Hemmings, 2025)).

Testes and seminal glomera were removed and cleaned in PBS, and total testis wet mass was measured on digital scales. Average total testis mass (combined left and right testes) was calculated for each species. Sperm samples were obtained by squeezing the distal end of the seminal glomerus. A volume of 2 µl of sperm were fixed in 50 µl of 5% formalin and later labeled with fluorescent cell labels (Hoechst 33342 and MitoTracker FM Green), before being photographed under a Leica DMLB fluorescence microscope with a darkfield filter. We aimed to photograph at least 10 sperm per individual, but the exact number varied depending on the availability of morphologically normal sperm in the sample, ultimately ranging from 6 to 10 sperm per male, averaging 10–30 sperm per species. Sperm photos were exported to ImageJ and measured to 0.01 µm. An average total sperm length was then calculated for each species. In total, we obtained oviduct samples from one female across 26 different species of Galliformes. For 20 of these species, we also collected testis (*n* = 51) and sperm samples (*n* = 500) from males. We obtained additional testis mass and sperm length data for the common pheasant (*Phasianus colchicus*) and Swinhoe’s pheasant (*Lophura swinhoii*) from Liao et al. (2019), resulting in a final sample size of 22 species for male traits (Figure 1B).

### Statistical analysis

All analyses were run in R V 4.3.2 R Core Team., 2023). Measurement repeatability, associated standard error, and 95% confidence intervals were calculated using the R package rptR (Stoffel et al., 2017). Sample ID was included as a random effect, with a Gaussian family and log link (SST length and area), or binary family and logit link (SST morphological categorizations), and 1000 parametric bootstraps. Measurements were considered highly repeatable at *R* > 0.7 with a *p-value* < .05 (Harper, 1994; Nakagawa & Schielzeth, 2010).

The shared evolutionary history of related species introduces non-independence between observations that must be controlled for in comparative analyses. We accounted for phylogenetic dependency using phylogenetic generalized least squares regression analyses (PGLS) and phylogenetic logistic regression (PLR), which incorporates the expected covariance between taxa using branch length data within a phylogeny. We used phylogenetic comparative techniques to explore the relationships between female reproductive traits (vagina length and sperm storage traits, including SST area, length, and morphology), sperm length, and post-copulatory sexual selection intensity. Relative testis mass is widely accepted as a reliable predictor of sperm competition intensity, but since we find relative vagina length to be highly correlated with relative testis mass (see the *Results* section), for subsequent analyses, we used relative vagina length as a more relevant proxy for post-copulatory sexual selection intensity inside the female reproductive tract. For models incorporating relative testis mass or relative vagina length, testis mass/vagina length and body mass were included as separate explanatory variables.

We obtained a Galliformes phylogeny with time-calibrated branch lengths from Stein et al. (2015), which was trimmed and combined with our data using the R packages *treeplyr* (Harmon, 2023) and *geiger* (Pennell et al., 2014) (Figure 1B). PGLS models incorporating continuous variables were performed using the R package *caper* (Orme et al., 2023), with the branch length transformation lambda (λ) estimated using maximum likelihood. Pagel’s λ values range from 0 to 1, with 0 indicating that trait (primarily the response variable) similarity is independent of phylogeny, while 1 indicates strong phylogenetic signal.

We evaluated models using a combination of diagnostic plot functions in *caper*, adjusted *R*^2^ values and *p-*values < .05. For the analyses incorporating tubule morphological categorizations, we used the R package *phylolm* (Ho & Ane, 2014) to perform a phylogenetically controlled logistic regression (i.e., for non-Gaussian data) (PLR) using the function *phyloglm*. For all models, continuous variables were log transformed, and female body mass (which had a strong phylogenetic signal (λ = 0.99, *p* = < .0001)), was included in every model as a fixed effect to account for allometric relationships (Figure 1B).

## Supplementary Material

qraf035_Supplemental_File

## Data Availability

All data and code are available in a Dryad Digital Repository, https://doi.org/10.5061/dryad.z08kprrrf (Assersohn & Hemmings, 2025).
