# Supplementary material for: Relative testis size is associated with vagina length but not sperm storage traits in Galliformes
Source: Evol Lett. 2025 Oct 3;9(6):686–95. doi: 10.1093/evlett/qraf035 (PMC12676457; doi:10.1093/evlett/qraf035)
Supplement: qraf035_Supplemental_File [file qraf035_supplemental_file.pdf]

Supplementary material for:

Relative testis size is associated with vagina length but not sperm storage traits in Galliformes

Katherine Assersohn and Nicola Hemmings
Corresponding author: Katherine Assersohn; k.assersohn@gmail.com

This file includes :
Supporting text
Figure S1
Table S1

Supporting text

Sperm storage structure analysis procedure

Following removal of the reproductive tract in female specimens, further dissection of the folds of the UVJ tissue took place in 2023. A full and detailed explanation of this process can be found in Assersohn et al., (2024), but briefly: UVJ samples were cut longitudinally and pinned open to reveal the internal luminal mucosa and mucosal folds of the UVJ. For each sample, 2 folds were dissected, and excess connective tissue was removed. Each fold was incubated for 5-10 min with 10-20µl of Hoechst 33342 dye (the exact volume used was dependent on the amount needed to fully submerge the fold). Hoechst was used to aid in visualisation of sperm in storage, and for clarifying the edges of sperm storage tubules (SSTs) against the surrounding tissue. The fold was then placed onto a slide, opened to lie flat (lamina propria side down), and a coverslip was added with Fluoromount-G^{TM} Aqueous Mounting Medium (Sigma-Aldrich). Once dry, slides were sealed with transparent nail varnish and stored in the dark until imaging.

Samples were examined under a fluorescence microscope, and the entire region containing the SSTs was imaged. In each field of view per sample, 5 SSTs were selected and measured using Image J. Measurements began at the tubule entrance, (which was clearly fluorescent against the surrounding tissue), as close to the first pixel representing clearly defined tubule tissue as possible, and ran along the midline of the tubule to the last pixel defining the end of the tubule against the surrounding tissue. Tubules often overlapped meaning we could not accurately identify every individual tubule, making counts of tubules, and true random

sampling unviable. Consequently, choice of tubules for measurement was made by haphazard sampling. Where possible (depending on the distribution of tubules), this was done by splitting the image into quadrants, choosing one tubule in each quadrant, and a tubule from the centre of the sample. Tubules were only chosen for measurement if the entire length from entrance to end was clearly discernible. While this may introduce a bias towards smaller tubules (because longer tubules are more likely to overlap), this was difficult to avoid in extremely dense samples. If fewer than 5 SSTs were present in the sample, then the total number of visible SSTs were measured. We considered branched tubules (i.e. tubules with one entrance but more than one 'blind end') to be one tubule of greater total length, rather than multiple shorter tubules. For a branched tubule, total length was therefore calculated as the sum of all branch lengths.

SST capacity was then calculated for each fold using particle analysis in ImageJ. The area of each tubule was traced using a Wacom pen and tablet (Wacom Co., Ltd.), after which the image was converted into black and white 8-bit. The image threshold was then adjusted to leave only the white filled outline of tubules against a black background. Particle analysis counts the total number of pixels contained within a selection and provides a measure of area. The area of tubule tissue (μm^2) was then calculated in this way for each image, providing the total area of tubule tissue coverage for each fold.

The location of SSTs varied between species, and while we consider every sample to contain the majority – and certainly the peak region – of SSTs, in some cases (6 samples) it appeared as if the tail end of the SST population may continue beyond the point of dissection at the vagina end (see Assersohn et al., (2024) for further details). Additionally, tubule morphology varied across the length of the sample (tubule size generally decreases towards the uterus), and sperm was never observed to be stored towards the uterus end of the fold, suggesting variation in the functional significance of SST structures along the sample. To account for these factors, we restricted our analyses of SST tissue area and length to the image within each fold that contained the highest density of tubule tissue, working under the assumption that the region of peak SST density is most likely to represent functional storage structures and therefore be the most comparable region of the UVJ across species.

SST length measurements and area calculations, and SST morphological categorisations, were repeated by a second observer on one randomly chosen image (from the pool of images

containing SSTs) for each fold sample. SST length measurements were repeated for the same subset of tubules measured originally, but also for an additional subset, to ensure that the method of haphazard SST choice was also repeatable. Individual repeatability in SST length and area was also calculated for the image of highest tubule density across both folds of the same sample and was found to be highly repeatable (see results in main text). Consequently, we considered it appropriate to calculate the mean SST length and area across both folds, providing us with one consolidating mean value for SST length and area per species.

Tubule morphological categorisations

Tubules were categorised based on their morphological appearance. We considered tubules to be one of 3 types: straight unbranched (simple, may bend but do not coil or branch), straight branched (generally possess between 1 and 3 long branches), or 'complex'. Complex tubules may be either branched, coiled, or agglomerate in appearance, according to the observations and categorisation criteria in Assersohn et al., (2024). The presence or absence (1/0) of a given tubule type was determined by whether a fold of the sample contained (1) or didn't contain (0) tubules of that type in any of the images along the fold.

General Observations from UVJ dissection

Tubules were highly variable in morphology along the length of the fold, and between species. Sperm were commonly observed stored within tubules towards the cloacal end of the fold, and generally within the region of highest tubule density, but stored sperm were unequally distributed among the population of tubules. Tubules usually decreased in size along the length of the fold towards the uterus end, by which point stored sperm were never observed, suggesting variation in SST function along the length of the fold. The position of tubules along the fold varied between species. In some cases, tubules were spread over a larger area and ended at the boundary of the uterus, whilst others did not approach the boundary of the uterus.

Repeatability

We found that SST length measurements were highly repeatable both within the same subset and across a different subset of tubules, indicating that haphazard SST choice was a reliable indicator of average SST length within an image. Repeatability was also high for SST area calculations, and the categorisation of straight unbranched tubules, straight branched

tubules, and tubule complexity (Table 1). Both average SST length and average SST area were repeatable across both folds within an individual (Table S1). Note that the data for Swinhoe's pheasant (*Lophura swinhoii*) was not incorporated in the repeatability analysis due to late inclusion in the dataset.

Table S1: Repeatability estimates (R- given on the link scale), with associated standard error (SE), confidence intervals (CI) and *P-values*.

	R	SE	CI	P
Measurement repeatability for SST area	0.75	0.07	0.59 - 0.84	< 0.0001
Measurement repeatability for SST length (same subsample)	0.98	0.01	0.97 – 0.99	< 0.0001
Measurement repeatability for SST length (different subsample)	0.81	0.05	0.70 - 0.89	< 0.0001
Categorisation repeatability for straight unbranched tubules	0.80	0.20	0.37 - 0.99	< 0.0001
Categorisation repeatability for straight branched tubules	0.93	0.15	0.94 - 1.00	< 0.0001
Categorisation repeatability for tubule complexity	0.95	0.01	0.95 - 1.00	< 0.0001
Within individual repeatability in SST area	0.83	0.07	0.69 - 0.92	< 0.0001
Within individual repeatability in SST length	0.84	0.07	0.66 - 0.93	< 0.0001

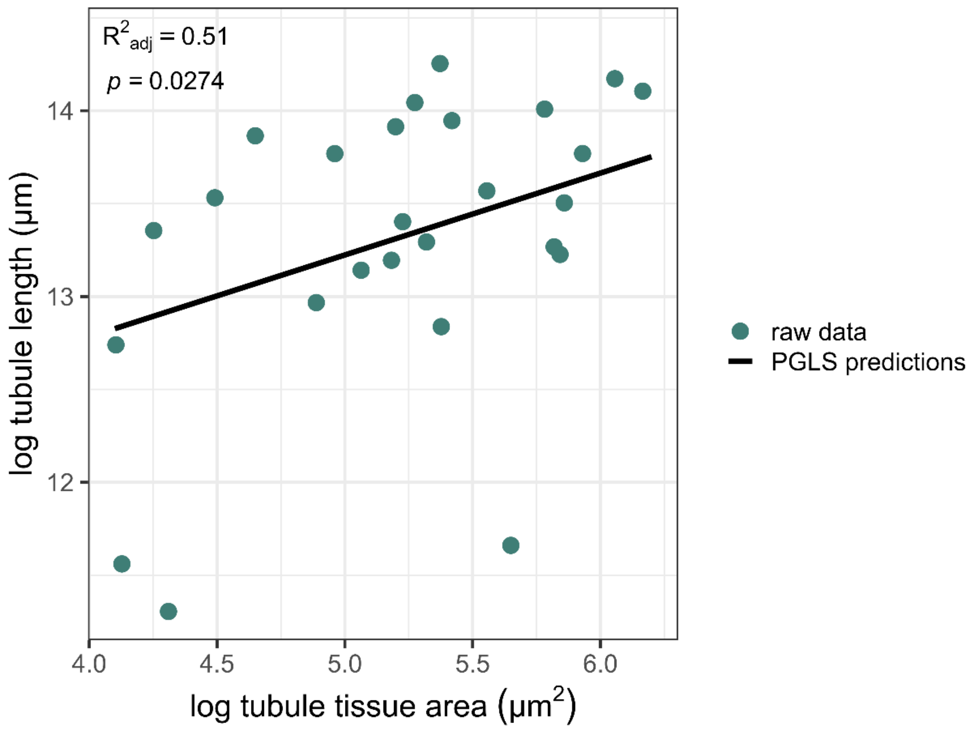

Figure S1: The relationship between average tubule length and species average tubule tissue (within the region of highest tubule density), across 26 species of Galliformes. Dots are the raw data points (each representing distinct species), and the solid line gives the predictions from the PGLS model. The PGLS model corrects for both phylogeny and body mass, but for the sake of plotting, predictions were calculated on data with body mass held constant at the mean to remove variation as a result of allometric relationships. Adjusted R^2 and p-values from the PGLS model are provided.
